# Response to “Clinical and radiological outcomes of long COVID and post-COVID fibrosis: Correspondence”

**DOI:** 10.5578/tt.20239923

**Published:** 2023-06-13

**Authors:** N. SARIOĞLU, G.D. AKSU, H. ÇOBAN, E. BÜLBÜL, G. DEMİRPOLAT, A. T. ARSLAN, F. EREL

**Affiliations:** 1 Department of Pulmonology, Balıkesir University Faculty of Medicine, Balıkesir, Türkiye; 2 Department of Radiology, Balıkesir University Faculty of Medicine, Balıkesir, Türkiye

Dear Editor

First of all, we would like to state that we greatly appreciate the interest in our study and thank you for your comments ( 1 ). Long -COVID is still a topic of debate. In our study, patients who were hospitalized for COVID pneumonia were evaluated clinically and radiologically at the 3^rd^ month ( 2 ). A positive SARS-CoV-2 nasopharyngeal swap on RT-PCR or a clinico-radioiogical diagnosis of COVID-19 was defined as COVID-19. The most common radiological finding in acute disease was bilateral ground glass areas (72.8%). The probability of undiagnosed comorbid conditions is very low in our cases. The old records of the patients were reviewed, and patients with suspected interstitial lung disease (n= 4) with a UIP pattern on an older Thorax CT before COVID were excluded. Cases with malignancies were excluded.

Haq and friends reported a case series of recurrent COVID-19 infection ( 1 ). The cases of recurrent COVID-19 infection presented here are also very interesting. These are patients who are younger than our patients (between 29-53 years) and mostly those who have mild to moderate illnesses and receive treatment at home. The time relapsed between being diagnosed with COVID-19 for the second time was 57 days in one case and 123-285 days in other cases. After the antibody response decreased (four months and later) they became ill again ( 1 ). All of our cases consisted of moderate-to-severe patients who were hospitalized. At the third month, most of the symptoms did not disappear completely, and complete recovery was not observed.

In a systematic review including 50 studies, 118 reinfected cases were reported ( 3 ). In these cases, the shortest reinfection period was reported as 19 days, and the longest was 293 days. Cough and fever were the most frequently reported symptoms in initial infections and reinfections. In our cases, no fever symptom was observed in any of the patients at the 3^rd^ month follow-up. We estimate this time to be short for reinfection, as the study included three months of follow-up. We did not detect any reinfected cases among our cases. In a cohort study in which serological measurements were made for approximately 30-152 days after SARS-CoV-2 infection, neutralizing antibody titers decreased an average of about 4-fold from 1 to 4 months after symptom onset ( 4 ).

Another issue is vaccination. In our cases, the rate of patients who had never been vaccinated was low. However, there were quite a few patients who did not have the booster dose, even though it was time, and had the disease. The period in which the disease reached its second peak in our country and variant strains increased was the autumn-winter period of 2020. Our study covers the follow-up period of hospitalized patients between December 2020 and March 2021. We observed that among the patients who applied during this period, elderly patients mostly preferred dead-virus vaccines and generally followed the vaccination schedule. We have seen that mRNA vaccines are applied more frequently in young people, but both the first vaccination rate and booster dose compliance are weak. Since it was not noted in the first application file on hospital admission, the exact numbers related to the vaccination status could not be reached. In individuals who have had the disease or have been vaccinated, the protective immune response may continue for months ( 5 ).

In conclusion, we believe that the innate immune response is very important and differs from person to person. In some people, the disease progresses severely and may cause long-term damage to the lungs.
